# Factors associated with the health status of childcare workers in southern Alberta, Canada

**DOI:** 10.1186/s13104-018-4039-5

**Published:** 2019-01-03

**Authors:** Oluwagbohunmi Awosoga, Afeez Abiola Hazzan, Suzanne McIntosh, Julia Dabravolskaj, Tolulope T. Sajobi, Jon Doan

**Affiliations:** 1 Faculty of Health Sciences, University of Lethbridge, 4401 University Drive West, Lethbridge, AB T1K 3M4 Canada; 2 Department of Healthcare Studies, The College at Brockport, State University of New York, 350 New Campus Drive, Brockport, NY 14420 USA; 3 Wellness & Recognition, Human Resources, University of Lethbridge, 4401 University Drive West, Lethbridge, AB T1K 3M4 Canada; 4 Edmonton Oliver Primary Care Network, 130, 11910 111 Ave NW, Edmonton, AB T5G 0E5 Canada; 5 Department of Community Health Sciences, University of Calgary, 2500 University Dr. NW, Calgary, AB T2N 1N4 Canada; 6 Kinesiology & Physical Education, Faculty of Arts and Science, University of Lethbridge, 4401 University Drive West, Lethbridge, AB T1K 3M4 Canada

**Keywords:** Childcare workers, Health status, Productivity, Retention, Quality

## Abstract

**Objective:**

There is growing evidence that the well-being of childcare workers has important implications for the care provided to children attending childcare centers. To add to the growing body of research in this area and to lay the groundwork for further research, we report the results of a pilot study examining factors that are associated with the health status of childcare workers in southern Alberta, Canada. The factors examined include: health control, employer’s interest in the childcare worker’s wellbeing, and actions that childcare workers are taking to improve their own health.

**Results:**

A total of 260 “Workplace Health and Risks Survey 2008” questionnaires were sent to 13 licensed daycare centers in southern Alberta, Canada. Of these, a total of 110 questionnaires were completed by childcare workers at these centers and returned. Regression analysis results show that *control over one’s health* (Standardized Beta = .504, p < .001), *employers’ knowledge of negative effects of stress* (Standardized Beta = − .328, p = .017), *employers’ interest in employees’ well*-*being* (Standardized Beta = .366, p = .008), and *actions that are planned to be taken to improve or maintain health in the future* (Standardized Beta = .231, p = .005) are all significant predictors of health status among childcare workers.

**Electronic supplementary material:**

The online version of this article (10.1186/s13104-018-4039-5) contains supplementary material, which is available to authorized users.

## Introduction

Childcare workers have to deal with a myriad of physical and emotional burden when caring for children, and this often comes at the expense of their own health [1]. Studies have shown that childcare workers are susceptible to a range of physical health concerns including musculoskeletal disorders and ergonomic risks [2]. These workers are at increased risk of infectious diseases like skin infections, head lice, and tuberculosis as a result of their job duties [3].

Research evidence shows that unfavorable health outcomes among childcare workers could be detrimental to both the workers and the children under their care [4, 5]. A recent Canadian study identified several factors that can potentially affect health outcomes among childcare workers including high physical and mental efforts, exposure to noise and bad odors, and inadequate remuneration [6]. A European study estimated the prevalence of effort-reward imbalance among childcare workers in Germany to be around 65% [7]. The lack of reciprocity between the efforts put in by childcare workers and the rewards (i.e. job security and promotion) received has been linked to a number of diseases including cardiovascular disease and depression [7].

Further, childcare workers face significant psychological challenges in the course of care provision. Factors that have been shown to engender stress among childcare workers include work conditions, events in their personal lives, and client factors such as age and care needs [8, 9]. However, research has shown that proper management of these issues could lead to better health outcomes for childcare workers, as well as favorable care outcomes (e.g. reduction in injuries) for children [6, 10].

Considering the wide spectrum of physical and psychological concerns prevalent among childcare workers as well as the potential benefits of addressing these issues, the current study aimed to: (1) identify factors associated with the health status of childcare workers in southern Alberta, Canada; and (2) define directions for further research.

## Main text

### Methods

This is a cross-sectional study conducted in Lethbridge, southern Alberta, Canada. Two hundred and sixty “Workplace Health and Risks Survey 2008” questionnaires were sent to 13 licensed daycare centers (Additional file 1) for their childcare workers to complete and return. The “Workplace Health and Risks Survey 2008” was developed by Health Canada and is available for public use. The survey addresses how people’s work can affect their health and includes the following sections: Rating your own health; feelings about my health and job; shift work; number of days absent from work due to illness, physical activity; worry, nerves or stress; sleep; seeking help; nutrition; someone to count on; smoking, alcohol, medication, and other drugs; safety; demographic information; and how your employer can help.

Of the 260 questionnaires sent out, a total of 110 were completed and returned to the research team (42.3% response rate). The study was approved by the Office of Research Ethics, University of Lethbridge, Alberta, Canada. Written informed consent was obtained from all participants. Each participant received a gift card worth $10 CAD as compensation for their time. In addition to descriptive statistics, correlation and regression analysis were conducted to determine the relationship between health status (dependent variable) of childcare workers and a range of variables including health control, employer’s interest in the childcare worker’s wellbeing, and actions that childcare workers are taking to improve their own health. Data was analyzed using IBM SPSS Statistics version 22.0 (IBM Corporation, Armonk, NY).

#### Description of variables

The list of variables examined include:

- *“Overall health state”*: In your opinion, would you say your health is?
- *“Health control”*: I am in control of my own health
- *“Employer’s knowledge”*: My employer knows that stress at work can have bad effects on employees’ health
- *“Employer’s sincere interest”*: My employer has a sincere interest in the well-being of its employees
- *“Threat to health”*: I do things that posed threat to my health
- *“Vigorous physical activity”*: In a typical week, how often do you spend at least 20 min a day in vigorous physical activity?
- *“Moderate physical activity”*: In a typical week, how often do you spend at least 30 min a day in moderate physical activity?
- *“Light physical activity”*: In a typical week, how often do you spend at least 60 min a day in light physical activity?
- *“Over*-*the*-*counter medication”*: In the last month, how often did you use over-the-counter medication or prescription drugs to help you sleep?
- *“Over*-*the*-*counter medication or prescription drugs for pain”*: In the last month, how often did you use over-the-counter medication or prescription drugs to reduce pain?
- *“Over*-*the*-*counter medication or prescription drugs for calming down”*: In the last month, how often did you use over-the-counter medication or prescription drugs to calm you down?
- *“Over*-*the*-*counter medication or prescription drugs for depression”*: In the last month, how often did you use over-the-counter medication or prescription drugs to relieve depression?
- *“Actions”*: Actions planned to take in order to improve or maintain health in the next year
- *“Smoking (Cigarette)”*: How many cigarettes do you smoke per day?”
- *“Smoking (Cigar)”*: How many cigar do you smoke per day?
- *“Alcohol”*: How many regular size (alcohol) do you drink per week?

### Results

#### Demographic characteristics

Table 1 presents the demographic characteristics of the childcare workers who responded to the survey. The majority of childcare workers in the study were aged 20 to 39 years old (70.3%). More than 48% had been working for their employers for 1–4 years, and around 25% had been on the same job for more than 5 years. Further, more than 60% had college diploma, while only 13.7% had a bachelor’s degree or higher. All of the participants had some certification in childcare.

**Table 1:** Demographic characteristics of childcare workers in the study

Variable name	Number (%)
Gender (N = 109)	
Male	1 (.92)
Female	108 (99.08)
Age of participants (N = 108), years	
Under 20	2 (1.9)
20–29	51 (47.2)
30–39	25 (23.1)
40–49	14 (13.0)
50–59	11 (10.2)
60 and older	5 (4.6)
Length of years with current employer (N = 109), years	
Less than 1	28 (25.7)
1–4	53 (48.6)
5–9	11 (10.1)
10–14	5 (4.6)
15 or more	12 (11.0)
Level of education (N = 110)	
No college diploma	27 (24.5)
College diploma (below bachelor level)	68 (61.8)
Bachelor’s degree or higher	15 (13.7)
Childcare certification level (N = 102)	
Child development assistance (1)	40 (39.2)
Child development worker (2)	19 (18.6)
Child development supervisor (3)	43 (42.2)

#### Health profile of childcare workers in the study

Table 2 shows the distribution of health, physical activity, over-the-counter medication use, and health habits among the childcare workers who participated in the study. Among the 110 participants, 8.7% estimated their health status (“*overall health state*”) to be “fair”, 39.8% indicated that it was “good”, 36.9% chose “very good”, and 14.6% estimated it to be “excellent”. Results also show that a total 63 (59.4%) participants (i.e. “disagree/strongly disagree”) did not think they do things at work that might pose a threat to their health, while 15 (14.2%) were unsure about this, and 28 (26.4%) agreed or strongly agreed with this statement (“*Threat to health*”).

**Table 2:** Distribution of health, physical activity, use of over-the-counter medication, and healthy habits among childcare workers

Variables	Excellent	Very good	Good	Fair	Poor	
Overall health state	15 (14.6%)	38 (36.9%)	41 (39.8%)	9 (8.7%)	0 (0%)	
	**Strongly agree**	**Agree**	**Not sure**	**Disagree**	**Strongly disagree**	
Health control	42 (38.2%)	54 (49.1%)	9 (8.2%)	5 (4.5%)	0 (0%)	
Employer’s knowledge	23 (21.7%)	53 (50.0%)	23 (21.7%)	4 (3.8%)	3 (2.8%)	
Employer’s sincere interest	32 (29.4%)	50 (45.9%)	16 (14.7%)	6 (5.5%)	5 (4.6%)	
Threat to health	7 (6.6%)	21 (19.8%)	15 (14.2%)	39 (36.8%)	24 (22.6%)	
	**Never**	**Once a week**	**2–3 times a week**	**4 times a week**	**5–6 times a week**	**Every day**
Vigorous physical activity	14 (12.7%)	31 (28.2%)	38 (34.5%)	10 (9.1%)	8 (7.3%)	9 (8.2%)
Moderate physical activity	7 (6.5%)	18 (16.8%)	43 (40.2%)	12 (11.2%)	12 (11.2%)	15 (14.0%)
Light physical activity	9 (8.3%)	16 (14.7%)	31 (28.4%)	11 (10.1%)	11 (10.1%)	31 (28.4%)
	**Daily or almost every day**	**2–3 times per week**	**Once a week**	**2–3 times per month**	**Once only**	**Not at all**
Use of over-the-counter medication for sleep	8 (7.3%)	4 (3.7%)	–	6 (5.5%)	5 (4.6%)	86 (78.9%)
Use of over-the-counter medication or prescription drugs for pain	4 (3.7%)	8 (7.4%)	9 (8.3%)	22 (20.4%)	9 (8.3%)	56 (51.9%)
Use of over-the-counter medication or prescription drugs for calming down	3 (2.8%)	1 (.9%)	–	3 (2.8%)	3 (2.8%)	99 (90.8%)
Use of over-the-counter medication or prescription drugs for depression	4 (3.7%)	–	–	1 (.9%)	1 (.9%)	101 (94.4%)
	**No actions (0–2) are planned**	**3–5 actions are planned**				
Actions	60 (54.5%)	50 (45.5%)				
	**None**	**Fewer than 10**	**10 or more**			
Smoking (Cigarette)	99 (91.7%)	5 (4.6%)	4 (3.7%)			
	**None**	**Fewer than 10**				
Smoking (Cigar)	106 (98.1%)	2 (1.9%)				
	**Zero**	**1 to 10**	**10 or more**			
Alcohol	86 (81.1%)	18 (16.9%)	2 (1.8%)			

Further, only 45.5% plan to take 3–5 actions (e.g. eat better, be more physically active, or get more or better sleep) in the upcoming year to improve their health status (“Actions”). Despite the very low percentage of childcare workers who are planning to take action to enhance or maintain their health, 87.3% of them considered themselves (i.e. “agree” or “strongly agree”) to be in control of their health. Among the participants, 12.7% and 28.2% did not exercise vigorously and exercised in this way only once a week respectively, while the majority (34.5%) exercised vigorously for 20 min 2–3 times a week. Majority (40.2%) of participants reported performing 30-min moderate exercises 2–3 times a week, and 28.4% of the participants reported taking 60-min light exercises on a daily basis. However, the percentages of those who are actively involved in physical activities are low for all three categories of exercises.

Further, 78.9% of the participants reported not taking medications to help them fall asleep, while 11% needed sleeping pills on a daily basis. In addition, the use of pain relief medication was reported, with 51.9% reporting not needing pain relief agents, while 48.1% needed this type of medication with different frequency (i.e. from daily to single use). For smoking habits, 91.7% and 98.1% of the participants did not smoke cigarettes and cigars respectively. In addition, 81.1% did not drink alcoholic beverages at all.

#### Correlation and regression analysis results

Health status was positively correlated with health control (r = .546, p < .001), employers’ interest in the well-being of employees’ (r = .248, p = .006), and actions that childcare workers are planning to take to improve their health (r = .341, p < .001). Additional correlation analyses were performed to examine the relationships between the included variables. Health control was positively associated with the employers’ knowledge about the negative effects of stress (r = .262, p = .004), employers’ interest in the well-being of employees’ (r = .227, p = .012); and the actions planned to be introduced in order to improve childcare workers’ health (r = .195, p = .026). Although these correlations are not strong, they are statistically significant. Further, employers’ knowledge of the negative effects of stress was positively associated with employers’ interest in the well-being of workers (r = .808, p < .001); and positively associated with the actions to improve or maintain health, although this was not statistically significant (r = .124, p = .110).

Simple linear regression analysis (adj.R^2^ = 37.7%, F(4,95) = 16.005, p < .001) was used to identify variables contributing to the health status of childcare workers (see Table 3). The regression analysis shows that *control over one’s health* (Standardized Beta = .504, p < .001), *employers’ knowledge of negative effects of stress* (Standardized Beta = − .328, p = .017), *employers’ interest in employees’ well*-*being* (Standardized Beta = .366, p = .008), and *actions that are planned to be taken to improve or maintain health in the future* (Standardized Beta = .231, p = .005) are all significant predictors of health status among childcare workers.

**Table 3:** Regression results for factors associated with the health status of childcare workers

Variables	Regression coefficients (B)	Standardized regression coefficients (β)	p-value
Health control	.532	.504	.000
Stress effect	− .302	− .328	.017
Wellness interest	.300	.366	.008
Actions	.390	.231	.005
Constant	.705		.102

R = .635, adjusted R^2^ = .377

### Discussion

The majority of childcare workers in this study classify their health status as either good, very good, or excellent, and this is consistent with results from other studies examining health status among childcare workers in similar settings [11]. The high percentage of workers who considered themselves to be in control of their health might indicate a different understanding of what health is, and what it means to be in control of one’s health. Thus, despite the high percentage of childcare workers who claim they had control over their health, only a few indicated having vigorous 20-min weekly exercise, while the majority answered that they have 60 min light exercises per day. However, the latter may have included physical activity while doing home-related work, rather than some extra physical activity. This indicates a need for programs directed toward adequate physical activity to be incorporated as part of injury prevention training programs among childcare workers.

As the majority of childcare workers do not plan to take action to improve or maintain their health in the next year, it is evident that these workers can benefit from the general healthy living recommendations. According to Baldwin et al. [11], 23.3% and 26.8% of childcare workers were overweight and obese respectively. Further, the 2004 Canadian Community Health Survey [12] reported that eating habits among Canadians in general are characterized by low consumption of vegetables and fruits, excess fat consumption, frequent eating at fast food restaurants, and unbalanced diet. Therefore, this issue may not be restricted to childcare workers.

Statistical analysis results showing positive correlations between health status, health control, and employer’s interest in childcare workers well-being highlight the important roles that employers play in ensuring healthy outcomes for their childcare workers. Taken together, these results indicate that childcare workers’ health status is better if they have more control over their health and take more action to improve it. Employers also play an important role, since the findings show that childcare workers’ health status is better when employers are interested in their employees’ well-being and have more knowledge about the effects of stress on their health. Therefore, it is important to educate employers on the health issues and burden associated with this line of work as this may encourage action (e.g. better health benefits) to improve the well-being of childcare workers.

### Conclusion

Childcare workers face enormous challenges in the course of caring for children, and this has important implications for their well-being and the quality of services provided to children. The current study identified factors that are associated with the health status of childcare workers in southern Alberta, Canada. Regression analysis results show that factors such as control over one’s health, employers’ knowledge of negative effects of stress, employers’ interest in employees’ well-being, and actions that are planned to be taken to improve or maintain health in the future are significant predictors of health status among childcare workers. This pilot study laid the foundation for further research on this topic.

## Limitations

Participant recruitment was focused on licensed childcare centers located in urban areas, and only 42.3% of the questionnaires mailed out were returned. The low response rate may be due to the busy schedule of childcare workers, as well as the stressful nature of the work. Further, day-home providers and agencies were not included in this pilot study. In addition, items on the questionnaire were not all related to the study, in which case future use of the findings will require an overall review of items on the questionnaire to support research questions. Finally, the psychometric properties of the questionnaire were unavailable. For future studies, it would be helpful to determine the total length of time participants have continued to work in daycare centres over the course of their life time versus the length of years with current employer. It would also be helpful to consider the diverse educational background and areas of specialization of childcare workers in licensed daycare centers.

## Additional file

**Additional file 1.** Workplace Health and Risks Survey 2008. This questionnaire focuses on the health status of childcare workers.
