# Supplementary material for: Factors associated with the health status of childcare workers in southern Alberta, Canada
Source: BMC Res Notes. 2019 Jan 3;12:4. doi: 10.1186/s13104-018-4039-5 (PMC6318857; doi:10.1186/s13104-018-4039-5)
Supplement: Supplementary file 1 — Additional file 1. Workplace Health and Risks Survey 2008. This questionnaire focuses on the health status of childcare workers. [file 13104_2018_4039_MOESM1_ESM.pdf]

What this Survey is About

Your work can affect your health. Most Canadians spend more than one-third of their waking hours at work. Workplace health programs can help employee and employer alike. After all, if we get healthier and have a healthy and supportive work environment, we not only feel better, but we can be more successful in our work and outside of work - and that benefits everybody.

Workplace health initiatives involve changing the workplace itself, the surroundings, workload, schedules, recognition or lines of communication to reduce stress or increase workplace safety. Workplace health programs also include stop-smoking programs, fitness program and employee assistance programs.

By answering the questions here, you are contributing to an overall picture of employee attitudes, needs and concerns. That way, your workplace health policies and programs can be based on real needs.

Your answers will be anonymous and kept in strict confidence. Do not put your name on this questionnaire. Once you fill it out and seal it in its envelope, it will never been seen by anyone at your workplace. An objective third party, who is a member of our research team, will count up the results and report to the research principal investigator who will in turn provide a report summary to interested participants, participating day care centres and day homes.

Instructions

- Please read each questions carefully and answer as accurately as you can, with reference to your own specific job and life. Your answers are completely anonymous and confidential.
- Use a pencil so you can erase any answer you want to change.
- When you are finished, seal your completed questionnaire in the attached envelope, make sure to fill out the entry form for the grocery gift card draw, and place the envelope and entry form in the drop box provided onsite.

Government Levels of Certification in Childcare

Level 1: Child Development Assistant

Level 2: Child Development Worker

Level 3: Child Development Supervisor

HEALTH STATUS OF CHILDCARE WORKERS IN SOUTHERN ALBERTA

Survey Questionnaire

Section A: A bit about you (please fill in appropriate answer)

Years of experience in childcare environment: _____________

Childcare certification level (circle one):

Child development assistant	Child development worker	Child development supervisor
Level I	Level II	Level III

Your work environment: Day care _____________ Day home _______________

Facility name (*optional*):____

Job Title: ____

Number of hours worked per day (*shift length; circle one*): 5 or less 6 7 8 or more

Number of children in your care (*circle one*):

1 to 6 7 to 12 13 to 19 20 to 26 27 and more

Age range of the children in your care (*circle one*):

0 to 12 months 1 to 2 years 3 to 4 years 5 to 6 years 7+ years

Number of staff in your classroom (*circle one*): 1 2 3 4 5+

Ethnicity (*optional; circle one*): Aboriginal Caucasian African American Asian Other

Would you be interested to receive a copy of the findings of the study (*circle*)? Yes No

If yes, please provide us with your contact information (*email preferred*):

<u>Important Note</u>

This questionnaire contains questions that are of a sensitive nature, and you are reminded that you can choose to not answer any of these questions.

<u>Survey Instrument</u>—Workplace Health and Risks Survey 2008 (by Health Canada—available for public use)

http://www.mentalhealthworks.ca/sites/default/files/1-WHPSP-Survey-eng.pdf

Completed surveys **<u>will not</u>** be submitted to Health Canada, only recorded and aggregated by University of Lethbridge researchers.

Section B: Rating your own health

1. **In your opinion, would you say your health is…..(*Circle one answer only.)***

Excellent Very good Good Fair Poor

PRODUCTIVITY

2. What, if anything, would you like to do in the next year to <u>improve or maintain your health?</u>

	ITEMS	Yes	No	N/A
01	Drink less coffee or tea	☐	☐	☐
02	Eat better	☐	☐	☐
03	Be more physically active	☐	☐	☐
04	Quit smoking, or smoke less	☐	☐	☐
05	Drink less alcohol	☐	☐	☐
06	Get more or better sleep	☐	☐	☐
	2.1 (*researcher use only*)			
07	Change jobs	☐	☐	☐
08	Change conditions of work	☐	☐	☐
09	Change my home situation	☐	☐	☐
10	Remove a major source of worry, nerves or stress from life	☐	☐	☐
	2.2 (*researcher use only*)			
11	Learn to cope better with worry, nerves or stress	☐	☐	☐
12	Learn to control anger better	☐	☐	☐
13	Learn to communicate better	☐	☐	☐
14	Learn to manage time better	☐	☐	☐
	2.3 (*researcher use only*)			
15	Get medical treatment	☐	☐	☐
16	Have my blood pressure checked	☐	☐	☐
17	Cut down on painkillers, anti-depressants, sleeping or calming medications	☐	☐	☐
18	Cut down on other medications	☐	☐	☐
	2.4 (*researcher use only*)			

3. What, if anything, is stopping you from making this change? (*Check all that apply to you.)*

	ITEMS	Yes	No	N/A
01	Don't know how to get started	☐	☐	☐
02	Not enough money	☐	☐	☐
03	Too much stress right now	☐	☐	☐
	3.1 (*researcher use only*)			
04	Problem isn't serious; there's no rush	☐	☐	☐
05	Too depressed	☐	☐	☐
06	It's too hard	☐	☐	☐
07	Don't want to change my ways	☐	☐	☐
08	Not sure I can really make a difference	☐	☐	☐
09	Fear of the unknown	☐	☐	☐
10	Lack of confidence	☐	☐	☐
	3.2 (*researcher use only*)			
11	No encouragement or help from employer			
12	No encouragement from family and friends			
	3.3 (*researcher use only*)			

ABSENTEEISM

4.1 In the last year, how many days in total were you away from work because you were sick?
(*from any cause; circle one***)**

01 0 1 to 5 6 to 9 10 to 19 20 and more

4.2 In the last year, how many days in total were you away from work because you were injured?
(*at work or at home; circle one***)**

01 0 1 to 5 6 to 9 10 to 19 20 and more

FEELINGS ABOUT MY HEALTH AND MY JOB

5. **Show how you feel about the following statements: (*Check one response for each statement.)***

ITEMS	Agree Strongly	Agree	Not Sure	Disa-gree	Disagree Strongly
**5.1** I am in control of my own health.	01 ☐	02 ☐	03 ☐	04 ☐	05 ☐
**5.2** I have an influence over the things that happen to me at work.	01 ☐	02 ☐	03 ☐	04 ☐	05 ☐
**5.3** I am satisfied with the fairness and respect I receive on the job.	01 ☐	02 ☐	03 ☐	04 ☐	05 ☐
**5.4** I fell I am well rewarded for the level of effort I put out for my job.	01 ☐	02 ☐	03 ☐	04 ☐	05 ☐
**5.5** I get as much out of my job as I put into it.	01 ☐	02 ☐	03 ☐	04 ☐	05 ☐
**5.6** At work, I feel I often have to do things or make decisions that I know are bad for my mental or physical health.	01 ☐	02 ☐	03 ☐	04 ☐	05 ☐
**5.7** On the whole, I like my job.	01 ☐	02 ☐	03 ☐	04 ☐	05 ☐
5.1 (*researcher use only*)					
**5.8** My employer know that stress at work can have bad effects on employees' health.	01 ☐	02 ☐	03 ☐	04 ☐	05 ☐
**5.9** My employer makes every effort to keep unnecessary stress at work to a minimum.	01 ☐	02 ☐	03 ☐	04 ☐	05 ☐
**5.10** I am satisfied with the recognition I receive from my employer for doing a good job.	01 ☐	02 ☐	03 ☐	04 ☐	05 ☐
**5.11** I am satisfied with the amount of involvement I have in decisions that affect my work.	01 ☐	02 ☐	03 ☐	04 ☐	05 ☐
**5.12** My employer has a sincere interest in the well-being of its employees.	01 ☐	02 ☐	03 ☐	04 ☐	05 ☐
**5.13** My employer provides some form of health care benefit to staff.	01 ☐	02 ☐	03 ☐	04 ☐	05 ☐
5.2 (*researcher use only*)					
**5.14** I think that, if I wanted to, I could quite easily find another job at least as satisfying as this one.	01 ☐	02 ☐	03 ☐	04 ☐	05 ☐
**5.15** If I had to find another job today, I think I would have all the skills and training necessary to do so.	01 ☐	02 ☐	03 ☐	04 ☐	05 ☐
**5.16** I look outside of my job for my main satisfaction in life.	01 ☐	02 ☐	03 ☐	04 ☐	05 ☐
5.3 (*researcher use only*)					

OTHER WORK

6. **1. Do you work for pay at a second job besides the one where you received this questionnaire?** ***(Check one answer only.)***

01 ☐ Yes, full-time   02 ☐ Yes, part-time   03 ☐ No

PHYSICAL ACTIVITY

7. Please answer the following questions as they apply to you during a typical week. *(Check one response only for each item.)*

ITEMS	Never	Less than once a week	1 to 3 times a week	4 times a week	5 or 6 times a week	Every day
**7.1 In a typical week, how often do you spend at least 20 minutes a day (in periods of at least 10 minutes each) in VIGOROUS LEISURE (not at work) physical activity?** [Vigorous physical activity results in a person feeling quite warm and out of breath from doing things such as aerobics, jogging, hockey, basketball, fast swimming, fast dancing, etc.]	01 ☐	02 ☐	03 ☐	04 ☐	05 ☐	06 ☐
**7.2 In a typical week, how often do you spend at least 30 minutes a day (in periods of at least 10 minutes each) in MODERATE LEISURE (not at work) physical activity?** [Moderate physical activity results in a person feeling warmer and breathing more quickly from doing such things as brisk walking, biking, raking leaves, swimming, dancing, water aerobics, etc.]	01 ☐	02 ☐	03 ☐	04 ☐	05 ☐	06 ☐
**7.3 In a typical week, now often do you spend at least 60 minutes a day (in periods of at least 10 minutes each) in LIGHT LEISURE (not at work) physical activity?** [Light physical activity results in a person starting to feel warm and breathing slightly more quickly from doing such things as light walking, volleyball, easy gardening, stretching, etc.]	01 ☐	02 ☐	03 ☐	04 ☐	05 ☐	06 ☐

WORRY, NERVES OR STRESS

8. What, if anything, caused you excess worry, "nerves" or stress at work in the last six months?

Check one response for each statement

ITEMS	Yes	No	N/A	ITEMS	Yes	No	N/A
01 I changed jobs	☐	☐	☐	16 Deadlines	☐	☐	☐
02 Too many changes within my job	☐	☐	☐	17 I don't enough feedback on how I'm doing	☐	☐	☐
03 Work hours are too long	☐	☐	☐	18 I don't get enough training	☐	☐	☐
04 Work hours are not flexible enough	☐	☐	☐	19 I'm not treated fairly here	☐	☐	☐
05 Balancing two or more jobs	☐	☐	☐	20 I'm afraid of being laid off	☐	☐	☐
06 Too much time pressure	☐	☐	☐	21 My work tires me physically	☐	☐	☐
07 Unscheduled overtime	☐	☐	☐	22 My work tires me mentally	☐	☐	☐
08 Having to bring work home too often	☐	☐	☐	23 My work is boring	☐	☐	☐
09 My duties are not clear	☐	☐	☐	24 I am being discriminated against	☐	☐	☐
10 My duties conflict with one another	☐	☐	☐	25 Conflict with other people work	☐	☐	☐
11 Management tries to control my work too much	☐	☐	☐	26 I feel isolated from peers, including co-workers	☐	☐	☐
12 I don't have enough influence over what I do and when I do it	☐	☐	☐	27 I have difficulty understanding written instructions	☐	☐	☐
13 Too much responsibility	☐	☐	☐	28 I don't have enough control over the pace of my work	☐	☐	☐
14 Too little responsibility	☐	☐	☐	29 Trying to cope at work with the results of an injury or illness	☐	☐	☐
15 Supervisors or managers have unrealistic expectations of me	☐	☐	☐				

9. What, if anything, caused you excess worry, “nerves” or stress <u>at home or outside of work</u> in the last six months? (Check one response for each statement).

ITEMS	Yes	No	N/A
01 A close family member or friend has been ill or injured	☐	☐	☐
02 A close family member or friend has died	☐	☐	☐
03 Unexpected pregnancy	☐	☐	☐
04 Trying to cope (outside work) with the results of own injury or illness	☐	☐	☐
9.1 (*researcher use only*)			
05 I have begun a new, close relationship (including getting married)	☐	☐	☐
06 Divorce or separation	☐	☐	☐
07 Arguments with my spouse, partner, children or roommates	☐	☐	☐
08 Arguments with other family or ex-family members	☐	☐	☐
9.2 (*researcher use only*)			
09 Physical abuse at home	☐	☐	☐
10 Verbal or emotional abuse at home	☐	☐	☐
11 Child care and/or elder care problems	☐	☐	☐
12 Child running away from home	☐	☐	☐
9.3 (*researcher use only*)			
13 Change in living situation (new roommate, family member leaving, etc.)	☐	☐	☐
14 Took on a big expense	☐	☐	☐
15 Took on a big loan	☐	☐	☐
16 I don’t have enough money	☐	☐	☐
17 I have too much to do	☐	☐	☐
18 Getting to and from work is difficult or takes too long	☐	☐	☐
9.4 (*researcher use only*)			

10. What, if anything, would you like to do to cope better with worry, "nerves" or stress? (Check one response for each statement)

ITEMS	Yes	No	N/A	ITEMS	Yes	No	N/A
01 Be more physically active	☐	☐	☐	09 Get out more often, make new friends, socialize	☐	☐	☐
02 Drink less coffee or tea	☐	☐	☐	10 Spend more time with my family	☐	☐	☐
03 Eat better	☐	☐	☐	11 Manage time better	☐	☐	☐
04 Sleep more or sleep better	☐	☐	☐	12 Learn more about coping with worry, "nerves" or stress	☐	☐	☐
10.1 (*researcher use only*)				13 Learn to relax	☐	☐	☐
05 Have more access to education and information	☐	☐	☐	14 Learn to control anger better	☐	☐	☐
06 Get more job skills	☐	☐	☐	15 Learn to communicate better	☐	☐	☐
07 Make a major change in my life (for example, change jobs, move or leave home)	☐	☐	☐	16 Improve the way I feel about how I look	☐	☐	☐
10.2 (*researcher use only*)				17 Get professional help	☐	☐	☐
08 I don't know what I could do	☐	☐	☐	10.3 (*researcher use only*)			

11. What, if anything, is stopping you from making these changes? Check one response for each statement.

ITEMS	Yes	No	N/A	
01 Problem isn't serious; there's no rush	☐	☐	☐	11.1 (*researcher use only*)
02 Too depressed	☐	☐	☐	
03 Don't know how to get started	☐	☐	☐	
04 It's too hard	☐	☐	☐	
05 Lack of self-confidence	☐	☐	☐	
06 Don't want to change my ways	☐	☐	☐	
07 Fear of the unknown	☐	☐	☐	
08 No encouragement from family and friends	☐	☐	☐	11.2 (*researcher use only*)
09 No encouragement or help from employer	☐	☐	☐	
10 Not sure I can really make a difference	☐	☐	☐	
11 I don't know what is stopping me	☐	☐	☐	

SLEEP

12. How many hours do you usually sleep every night (*or day, if on shift work; circle one*)?

01 5 or less 6 to 7 ¾ 8 or more

---

13. How often do you have trouble sleeping? (*Check one answer only*)

01 ☐ More than once a week

02 ☐ Once a week or less

03 ☐ Never

---

14. In general, how often are you so physically or mentally tired at the end of work that you do not really enjoy your time away from work? (*Check one answer only*)

01 ☐ Very often

02 ☐ Often

03 ☐ Not very often

04 ☐ Never

SEEKING HELP

15. During the last year, did you seek help or counselling for a non-medical, personal or emotional problem of any kind? (*Check one answer only*)

01 ☐ Yes, through my employer or through a service provided by my employer (such as an employee assistance program)

02 ☐ Yes, but not through my employer

03 ☐ No, but I thought about it

04 ☐ No

NUTRITION

16. What, if anything, would you like to do in the next year to improve how, when, what or how much you eat? (*Please check one response for each statement*)

ITEMS	Yes	No	N/A
01 Eat more vegetables and fruit	☐	☐	☐
02 Drink more water	☐	☐	☐
03 Eat breakfast more often	☐	☐	☐
16.1 (*researcher use only*)			
04 Take time to eat	☐	☐	☐
05 Choose smaller portions on foods	☐	☐	☐
06 Cut back on junk foods	☐	☐	☐
07 Limit foods and beverages high in calories, fat, sugar or salt	☐	☐	☐
08 Follow Canada's Food Guide recommendations	☐	☐	☐
16.2 (*researcher use only*)			
09 Learn more about health eating (nutrition)	☐	☐	☐
10 Consult nutrition labels on food products more often	☐	☐	☐
16.3 (*researcher use only*)			

17. What, if anything, is stopping you from improving how, when, what or how much you eat? (*Please check one response for each statement*)

ITEMS	Yes	No	N/A
01 Limited choices in the cafeteria or in eating places near where I work	☐	☐	☐
02 Job pressures, job schedule	☐	☐	☐
03 Expense (healthy foods cost more)	☐	☐	☐
17.1 (*researcher use only*)			
04 Find it hard to eat well when I eat out	☐	☐	☐
05 Too much stress at home	☐	☐	☐
06 Dislike idea of dieting	☐	☐	☐
17.2 (*researcher use only*)			
07 Don't know what is stopping me	☐	☐	☐

SOMEONE TO COUNT ON

18. Of the people you <u>know right now</u>, who would really <u>listen to you carefully and sympathetically</u> if you were seriously upset about something? (*Please check one response for each statement*)

ITEMS	Yes	No	N/A
01 One or more co-workers	☐	☐	☐
02 An EAP (Employee Assistance Program) or EFAP (Employee and Family Assistance Program) counsellor	☐	☐	☐
03 My boss	☐	☐	☐
18.1 (*researcher use only*)			
04 My spouse or partner	☐	☐	☐
05 One or more other family members	☐	☐	☐
06 One or more close friends	☐	☐	☐
18.2 (*researcher use only*)			
07 A clergyman, rabbi or another religious official	☐	☐	☐
08 A lawyer	☐	☐	☐
09 One or more neighbours	☐	☐	☐
10 One or more people in my church, synagogue, etc.	☐	☐	☐
18.3 (*researcher use only*)			
11 Telephone help line	☐	☐	☐
12 No one	☐	☐	☐

18.13 Do you have children for whom you are wholly or partly responsible?

01 ☐ Yes

02 ☐ No

18.14 Do you have other people (like elderly parents) for whom you are wholly or partly responsible?

01 ☐ Yes

02 ☐ No

SAFETY

19. Below is a list of health and safety hazards and unpleasant working conditions. Please indicate the ones about which you are <u>very concerned</u> in <u>your</u> workplace by checking the relevant boxes below.

ITEMS	Yes	No	N/A
01 Too much heat or cold	☐	☐	☐
02 Bad air (stuffy, not enough air, etc.)	☐	☐	☐
03 Too much noise or vibration	☐	☐	☐
04 Poor work space, not enough work space, changing work space	☐	☐	☐
05 Poor lighting (too much, too little, etc.)	☐	☐	☐
06 Litter or mess in work area	☐	☐	☐
07 Slipping and tripping	☐	☐	☐
08 Infectious diseases	☐	☐	☐
09 Child-sized furniture and finishings	☐	☐	☐
10 Lack of facilities or access for employees with disabilities	☐	☐	☐
11 Risk of physical strain (e.g., back, wrist, neck, etc.)	☐	☐	☐
12 Not enough safety training	☐	☐	☐

20. What would you do if your supervisor told you to do something that you thought was dangerous for your health and safety? (*Check one response only*)

01 ☐ I would do it anyway and not complain to anyone in authority

02 ☐ I would do it, but complain to someone in authority later

03 ☐ I would not do it until I was satisfied that there was no danger

04 ☐ I am not sure what I would do

YOUR BACKGROUND

In order to make sense of the information you have given us so far, we need to ask a few personal questions. Your answers will help us figure out which groups have what needs. Please remember, though, that no one will use it to identify you.

21. How old are you?

01 ☐ Under 20	07 ☐ 45 to 49
02 ☐ 20 to 24	08 ☐ 50 to 54
03 ☐ 25 to 29	09 ☐ 55 to 59
04 ☐ 30 to 34	10 ☐ 60 to 64
05 ☐ 35 to 39	11 ☐ 65 to 69
06 ☐ 40 to 44	12 ☐ 70+

---

22. What is your marital status right now? (*Check one answer only.)*

01 ☐ Single/never married

02 ☐ Married

03 ☐ Widowed

04 ☐ Separated

05 ☐ Divorced

06 ☐ Living with someone

23. What is your sex?

01 ☐ Male

02 ☐ Female

24. How long have you been with your current employer? ***(****Check one answer only****)***

01 ☐ Less than 1 year

02 ☐ 1 - 4 years

03 ☐ 5 - 9 years

04 ☐ 10 - 14 years

05 ☐ 15 or more years

25. What is your level of education? ***(****Check the one answer that most closely reflects the highest education level you have reached****)***

01 ☐ Went to secondary/high school but didn't finish

02 ☐ Secondary/high school graduation certificate or equivalent

03 ☐ Went to community college, etc. but did not finish

04 ☐ Diploma or certificate from community college in Early Childhood Education

05 ☐ Went to university but didn't finish

06 ☐ University certificate or diploma below bachelor level

07 ☐ Bachelor's degree (e.g., B.A., B.Sc., LL.B.)

08 ☐ University certificate or diploma above bachelor level including Master's degree (e.g., M.A., M.Sc., M.Ed.) or professional degree (e.g., Degree in medicine, dentistry, veterinary medicine or optometry (M.D., D.D.S., D.M.D., D.V.M., O.D.) or professional designation (CGA, etc.) or earned doctorate (e.g., Ph.D., D.Sc., D.Ed.)

Specify your area of specialization (Major) ________________________

HOW YOUR EMPLOYER CAN HELP

26. How do you think your employer could help you improve your health? ***(Check all the items that you think would be helpful to you personally.)***

ITEMS	Yes	No	N/A
01 Provide (better) health benefits	☐	☐	☐
02 Get more employee input on how work is done here	☐	☐	☐
03 Introduce or extend flexible hours	☐	☐	☐
04 Provide more workplace health and safety training	☐	☐	☐
05 Train supervisors or managers to be more sensitive to employees' concerns	☐	☐	☐
06 Communicate more openly with employees	☐	☐	☐
07 Provide (better) employee assistance programs to help people get counselling on personal, financial or other problems	☐	☐	☐
08 Provide or support child care	☐	☐	☐
09 Look at how current shift schedules affect employees' sleep and health	☐	☐	☐
10 Support use of external fitness facilities by helping with cost	☐	☐	☐
11 Provide or support stress control program	☐	☐	☐
12 Provide or support other programs that will improve employees' health	☐	☐	☐
13 Provide or support more social/family events	☐	☐	☐
14 Encourage employees to spend time improving their health	☐	☐	☐

WE WILL APPRECIATE IT IF YOU CAN COMPLETE PART 2 OF THIS SURVEY

PART TWO ASK QUESTIONS ON WORK ACTIVITY
